# Handshake stewardship over a decade: refinement after successful implementation

**DOI:** 10.1017/ash.2026.10863

**Published:** 2026-09-25

**Authors:** Kira E. Frappa, Matthew J. Weber, Kelly Pearce, Sarah K. Parker

**Affiliations:** 1 Department of Pharmacy, Children’s Hospital Colorado, Aurora, CO, USA; 2 Section of Pediatric Infectious Diseases, University of Colorado School of Medicine, Aurora, CO, USA; 3 Infection Prevention, Children’s Hospital Colorado, Aurora, CO, USA

## Abstract

**Objective:**

To assess anti-infective use after over a decade of handshake stewardship by analyzing days of therapy per thousand patient days for all anti-infectives.

**Design:**

Retrospective evaluation pre- and post-implementation of handshake stewardship from October 2010 to September 2025.

**Setting:**

A pediatric hospital in Aurora, Colorado, with a licensed bed capacity of 472 beds. Handshake stewardship at our institution began in October 2013, after two years of planning (October 2011–September 2013); prior, there was no form of stewardship (October 2010–September 2011).

**Patients:**

All hospitalized patients administered anti-infectives during the study period.

**Methods:**

Days of therapy per thousand patient days were extracted from the electronic medical record and stratified by unit, medication, and route of administration. Antibacterial agents were grouped by National Healthcare Safety Network Antimicrobial Use and Resistance Module categories for pediatric resistant Gram-positive and hospital-onset infections. Yearly medians were utilized to define a refinement phase.

**Results:**

During 12 years of handshake stewardship (2013–2025), anti-infective use hospital-wide declined 33%. A maintenance phase, when refinement in anti-infective use was observed though overall use plateaued numerically, began in October 2021.

**Conclusions:**

The goal of stewardship is judicious use, not no use. We describe the maturation of a stewardship program over 12 years, demonstrating that handshake stewardship is both effective and sustainable, and that over time the focus shifts from decreased overall days of therapy per thousand patient days towards optimal use, reflected in fine-tuned use of specific agents.

## Introduction

Antimicrobial stewardship programs (ASPs) are essential entities within the healthcare system to help curb injudicious use of anti-infectives. ASPs employ several strategies to optimize anti-infective use to avoid adverse effects such as *Clostridioides difficile* infections and development of antibiotic resistance. In 2019, the Centers for Disease Control and Prevention (CDC) updated the Core Elements for Hospital Antibiotic Stewardship Programs.^1^ The newest version of the Core Elements highlights priority interventions such as prospective audit and feedback (PAF) and preauthorization. These interventions comprise the core components of any stewardship program. In addition, the Joint Commission (TJC) R3 Reports include antibiotic stewardship requirements. One of the 2023 TJC elements of performance (EP), EP 16, states that “The antibiotic stewardship program monitors the hospital’s antibiotic use by analyzing data on days of therapy [DOT] per 1,000 days present or 1,000 patient days [PD]…”.^2^

PAF is demonstrated to improve antibiotic use and reduce antibiotic resistance throughout hospitals.^3^ Handshake stewardship is an expanded form of PAF mentioned in both CDC guidance and TJC best practices.^1,4^ This method was first started by our hospital in 2013, evaluated at one and five years, and continues to be our preferred method.^5,6^

There are no publications analyzing long-term sustainability of a handshake stewardship program and resilience over time in the context of the evolution of the healthcare landscape. We now have over 12 years of experience with handshake stewardship, and our program has evolved to address new challenges. It is natural that a stewardship program tackle “low hanging fruit” first, curbing anti-infective use relatively quickly, and then move on to “high hanging fruit” where focus shifts from decreasing use to refining use (a refinement period).^7^ The aim of this study is to describe trends in hospital-wide anti-infective use for the 12-year handshake stewardship era, particularly during the refinement phase, expanding beyond previously published data at our institution.

## Methods

### Setting

Children’s Hospital Colorado (Children’s Colorado) is a free-standing, nonprofit pediatric hospital serving a seven-state region. In 2025, we had 15,566 inpatient admissions with a licensed bed capacity of 472 beds at the Anschutz Medical Campus, located in Aurora, Colorado.^8^ Units include the cardiac intensive care unit (CICU)/cardiac progressive care unit (CPCU) (56 beds), neonatal intensive care unit (NICU) (82 beds), pediatric intensive care unit (PICU) (32 beds), surgery/rehabilitation (91 beds), hematology/oncology/bone marrow transplant (BMT) (48 beds), and general medicine units (115 beds). Transplants provided include heart, liver, kidney, and bone marrow, as well as immunotherapy. Combined heart, liver, and kidney transplant numbers increased from a median of 35/year in 2014 (first full year of handshake stewardship) to 52/year since 2015; liquid transplant (bone marrow and CAR-T) numbers remained steady at a median of 74/year from 2015 (first year available) to 2025. The case-mix index (All Patient Refined Diagnosis Related Groups Weight/number of patient discharges) was 1.43 in 2014 and 1.58 in 2025.

Throughout the study period, the time involved and breakdown of handshake stewardship rounds remained similar to previous reports: two stewards reviewed the 24- and 48–72 hour time durations orders, collaborated with the Microbiology, Infection Control, and Infectious Diseases teams, and rounded in person.^5,6^ During the week, one physician and one pharmacist rounded daily and on weekends (added in 2023), one pharmacist completed ASP review and rounds. The process required approximately four hours per day, per steward. Anti-infective review included all orders for antibacterials, antifungals, and antivirals via three routes (intravenous, enteral, and inhaled). We do not have prior authorization or use rules-based software.

While the process is largely unchanged, the number of personnel splitting stewardship days expanded since our previous publications.^5,6^ As of 2025, we had six pediatric Infectious Diseases physicians (1.2 shared FTE), three full-time pediatric Infectious Diseases pharmacists, and two PRN pharmacists who all shared antimicrobial stewardship responsibilities.

### Study design

Hospital-wide anti-infective use was retrospectively measured from October 2010 through September 2025, which encompasses pre and postimplementation of handshake stewardship. The preimplementation period lasted from October 2010 to September 2013 and included a year of prestewardship and two years of planning. Planning spanned October 2011–September 2013, during which clinical pathways were developed, and the handshake stewardship methodology was designed. Finally, data from postimplementation, the handshake stewardship (HS, followed by year 1–12) era, started October 2013 (HS1) until September 2025 (HS12).

Days of therapy per 1,000 patient days (DOT/1,000 PD) were used to measure anti-infective use. PD were based on midnight census. DOT for anti-infectives were extracted from the electronic medical record reporting database, aggregated, and analyzed in Tableau (*Salesforce, Inc; Server Version 2025.1.6*). The Tableau application includes hospital-wide DOT/1,000 PD stratified by unit, medication, indication, and route of administration. A DOT was defined as the receipt of at least one dose of an anti-infective on a single calendar day, with each anti-infective counted separately.^9^ Patients from Maternal Fetal Medicine/Obstetrics and Psychiatric/Behavioral Science services were excluded from both numerator and denominator calculations. Our methodology was the same as previous publications.^5,6^ Of note, DOT/1,000 PD in the current publication do not match the numbers in the previous publications due to differences in patient day methodology (primarily census count methodology) between our legacy approach and the newer enterprise-standard methodology, though the overall trends are similar. We therefore recalculated all data back through the prestewardship era (2010) using the updated methodology to ensure consistency across all phases of the study.

Our groupings for pediatric antibacterial agents for resistant Gram-positive infections (GP-MRSA) and broad-spectrum agents for hospital-onset infections (BSHO) were based on the Standardized Antimicrobial Administration Ratio (SAAR) antimicrobial agent categories set by the National Healthcare Safety Network Antimicrobial Use and Resistance Module.^9^ SAAR agents with zero use are not listed. Antibacterials with high bioavailability were determined based on our internal policy for intravenous to enteral switch and included ciprofloxacin, clindamycin, doxycycline, levofloxacin, linezolid, metronidazole, rifampin, and sulfamethoxazole/trimethoprim. Antimicrobial costs were determined utilizing the Children’s Colorado Epic Cost Report and are calculated as quantity (eg, grams, units) medication administered x average wholesale price (AWP). This study was approved as quality improvement by the Children’s Colorado Organizational Research Risk and Quality Improvement Review Panel.

### Statistical analysis

A negative binomial model was fit to the anti-infective DOT count data per PD by month accounting for overdispersion, and a percent change per month with 95% confidence interval was derived from an incidence rate ratio to quantify the change over time. The same methods were applied to measure trends in the proportion of intravenous and oral antibiotic use with antibiotic days as the denominator. Reported p-values are obtained from the model coefficient, and values below 0.05 are considered significant, suggesting a non-zero trend. Absolute and percent change represent the difference in point estimate of the trend model from the first day of October 2013 to the last day of September 2025 (HS1–HS12). Yearly medians were used to visualize the plateau (refinement) phase in the DOT/1,000 PD trend. Data beyond the identified refinement phase were reviewed individually to evaluate change in antimicrobial usage leading to the stabilization of DOT rate. RStudio was used for data analysis (*Posit Software, PBC, Boston, MA; R version 4.4.0)*.^10^

## Results

### Stewardship workload

The median number of patients/orders reviewed daily increased from 1,269 patients/1,718 orders monthly in HS1 to 1,825 patients/2,378 orders in HS12; the percentage of orders resulting in an intervention remained stable at 8.7%, thus there was one intervention for every 11.4 antimicrobial orders reviewed. The acceptance rate remained steady at 88.5%.

### Overall anti-infective use

DOT/1,000 PD were evaluated across the preimplementation period (3 yrs) and 12 years of handshake stewardship era (Figure 1). For the 12 years (October 2013 (HS1)–September 2025 (HS12)) of handshake stewardship, anti-infective use hospital-wide steadily declined 33.0% (1,001.2–670.9 DOT/1,000 PD), with an estimated −0.28% slope change per month (*P* < .001). Antibacterial use decreased 29.8% (776.1 to 545.1, *P* < .001) from HS1 to HS12. Antifungal and antiviral use decreased 59.4% (145.2–59.0, *P* < .001) and 16.0% (82.2–69.1, *P* = .0044), respectively from HS1 to HS12.

**Figure 1.**
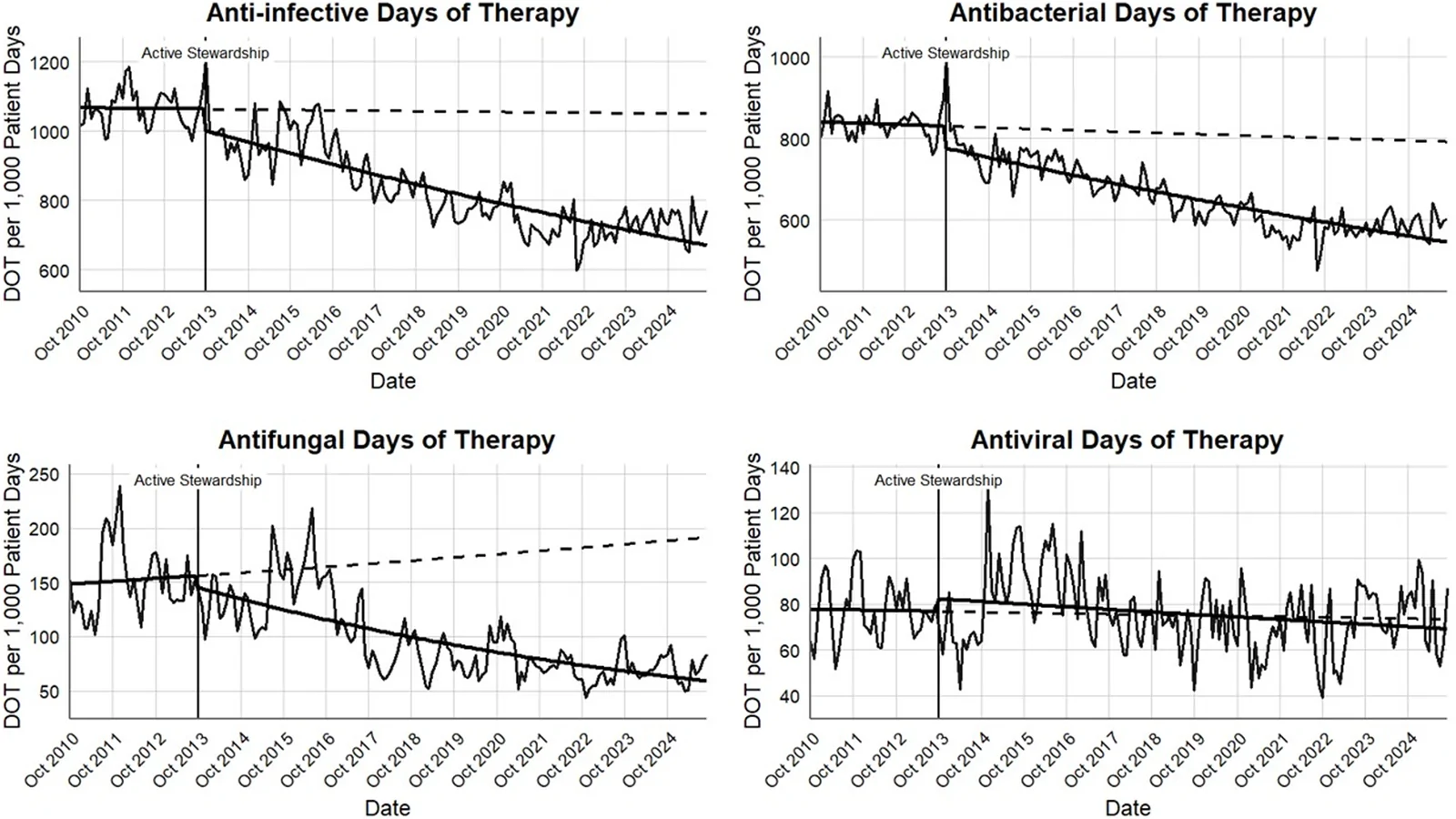
Figure 1 long description. Days of therapy per 1,000 patient days from October 2010 (preimplementation) to September 2025 (postimplementation, handshake stewardship era). *Note:* DOT, days of therapy. Solid line represents the actual trend in pre-/post-implementation of handshake stewardship. Dotted line represents the projected trend for the pre-implementation period if handshake stewardship had not been implemented.

### Individual antibacterial agents

Hospitalwide antibacterial use decreased significantly (*P* < .05) for all individual agents except ceftriaxone, linezolid, and the antipseudomonal cephalosporins (Table 1). Ceftriaxone use remained stable during the stewardship period (−0.09%, *P* = .984). Linezolid significantly increased (+778.1%, *P* < .001). Antipseudomonal cephalosporins as a group increased; driven by increases in ceftazidime use (+16.9%, *P* = .0028).

**Table 1.** Anti-infective use during handshake stewardship era (October 2013 (HS1)–September 2025 (HS12)) by medication grouping, individual agents, and units Table 1 long description.

	Stewardship slope change per month, % (95% CI)	Regression coefficient *P*-value	Regression estimated absolute and percent change	Point estimates first to most recent year, DOT/1,000 PD
October 2013–September 2025; Point estimates done on first year (2013) and last year (2025)				
**HOSPITAL-WIDE**				
Anti-infectives	−0.28% (−0.31%, −0.25%)	<.001*	−330.3 (−33.0%)	1,001.2–670.9
Antibacterials	−0.25% (−0.27%, −0.22%)	<.001*	−230.9 (−29.8%)	776.1–545.1
Antifungals	−0.63% (−0.72%, −0.54%)	<.001*	−86.2 (−59.4%)	145.2–59.0
Antivirals	−0.12% (−0.21%, −0.04%)	.00443*	−13.1 (−16.0%)	82.2–69.1
Vancomycin	−0.48% (−0.53%, −0.42%)	<.001*	−46.3 (−49.6%)	93.4–47.1
Linezolid	+1.53% (1.30%, 1.76%)	<.001*	+13.6 (+778.1%)	1.75–15.4
Clindamycin	−1.71% (−1.85%, −1.58%)	<.001*	−48.8 (−91.6%)	53.3–4.5
Carbapenems (ertapenem, meropenem)	−1.38% (−1.55%, −1.21%)	<.001*	−37.8 (−86.3%)	43.8–6.0
Fluoroquinolones (ciprofloxacin, levofloxacin)	−0.38% (−0.48%, −0.29%)	<.001*	−14.7 (−42.1%)	34.9–20.2
Ceftriaxone	0.00% (−0.06%, 0.06%)	.984	−0.05 (−0.09%)	54.2–54.1
Antipseudomonal cephalosporins (ceftazidime and cefepime)	+0.11% (0.03%, 0.19%)	.00282*	+12.7 (+16.9%)	74.6–87.3
Piperacillin-tazobactam	−0.40% (−0.64%, −0.17%)	.00236*	−3.6 (−43.9%)	8.1–4.6
**CICU/CPCU**				
All antibacterials	−0.14% (−0.21%, −0.08%)	<.001*	−124.0 (−18.4%)	674.9–550.9
GP-MRSA^a^	−0.53% (−0.66%, −0.41%)	<.001*	−81.6 (−53.3%)	153.1–71.5
BSHO^b^	−0.13% (−0.27%, 0.02%)	.0706	−15.4 (−16.8%)	91.6–76.2
**NICU**				
All antibacterials	−0.24% (−0.30%, −0.18%)	<.001*	−137.1 (−29.3%)	467.9–330.8
GP-MRSA^a^	−0.31% (−0.43%, −0.19%)	<.001*	−24.4 (−36.1%)	67.7–43.3
BSHO^b^	+0.99% (0.83%, 1.14%)	<.001*	+109.0 (+306.3%)	35.6–144.6
**PICU**				
All antibacterials	−0.27% (−0.32%, −0.23%)	<.001*	−439.0 (−32.3%)	1,358.4–919.4
GP-MRSA^a^	−0.45% (−0.53%, −0.37%)	<.001*	−158.9 (−47.3%)	335.9–177.0
BSHO^b^	−0.23% (−0.34%, −0.13%)	<.001*	−83.3 (−28.3%)	294.9–211.5
**SURGERY/REHABILITATION**				
All antibacterials	+0.01% (−0.04%, 0.05%)	.685	+8.2 (+1.3%)	624.9–633.1
GP-MRSA^a^	−0.21% (−0.32%, −0.10%)	<.001*	−24.1 (−25.8%)	93.1– 69.1
BSHO^b^	−0.16% (−0.31%, −0.01%)	.0424*	−13.0 (−20.2%)	64.7–51.6
**HEMATOLOGY/ONCOLOGY/BMT**				
All antibacterials	−0.09% (−0.13%, −0.05%)	<.001*	−126.4 (−12.1%)	1,045.9–919.6
GP-MRSA^a^	−0.10% (−0.22%, 0.03%)	.128	−17.9 (−12.8%)	140.5–122.6
BSHO^b^	−0.14% (−0.19%, −0.09%)	<.001*	−95.3 (−18.1%)	526.9–431.7
**GENERAL MEDICINE**				
All antibacterials	−0.47% (−0.52%, −0.42%)	<.001*	−417.1 (−49.1%)	848.9–431.8
GP-MRSA^a^	−0.96% (−1.06%, −0.86%)	<.001*	−105.2 (−74.8%)	140.7–35.5
BSHO^b^	−1.08% (−1.20%, −0.97%)	<.001*	−184.8 (−78.9%)	234.2–49.4

DOT, days of therapy; PD, patient days; CICU/CPCU, cardiac intensive care unit/cardiac progressive care unit; GP-MRSA, agents for resistant gram-positive infections in pediatrics; BSHO, agents for hospital-onset infections in pediatrics; NICU, neonatal intensive care unit; PICU, pediatric intensive care unit; BMT, bone marrow transplant.

* Significant *P*-value (<.05).

a Ceftaroline, ceftobiprole medocaril, clindamycin, dalbavancin, daptomycin, linezolid, oritavancin, tedizolid, telavancin, and intravenous vancomycin.

b Intravenous amikacin, intravenous aztreonam, cefepime, ceftazidime, ciprofloxacin, delafloxacin, ertapenem, imipenem/cilastatin, levofloxacin, meropenem, moxifloxacin, piperacillin/tazobactam, and intravenous tobramycin.

### Antibacterial groupings (GP-MRSA and BSHO) by clinical units

Antibacterial use in all units decreased significantly over the 12-year postimplementation period, except for the surgery/rehabilitation unit, which experienced non-significant change (+1.3%, *P* = .685) in DOT/1,000 PD (Table 1). GP-MRSA agents also decreased significantly on all units except hematology/oncology/BMT, where use decreased but not significantly (−12.8%, *P* = .128). In the NICU, use of BSHO agents increased significantly (+306.3%, *P* < .001).

### Enteral and intravenous routes of administration

In HS1, an estimated 29.7% of antibiotic days were enteral and 68.9% were intravenous. In HS12, enteral administrations increased (+14.6%, *P* < .001) to an estimated 34.0% and parenteral decreased (−5.0%, *P* < .001) to 65.5%. Isolating the highly bioavailable medications, in HS1, an estimated 58.9% were enteral and 41.0% were parenteral. In HS12, highly bioavailable enteral administrations changed non-significantly to an estimated 59.0% (+0.2%, *P* = .93) and parenteral to 40.9% (−.25%, *P* = .949).

### Phases over time

Our overall DOT reached a nadir starting in October 2021 (HS9, identified by the lowest yearly medians; a run chart depicts the antibacterial change over time while also highlighting major milestones within the history of the program Figure 2). In addition to these milestones, we continued standard stewardship tasks including pathway and guideline creation/updates. The post2021 plateau we view as a “refinement phase” (HS8–HS12).

**Figure 2.**
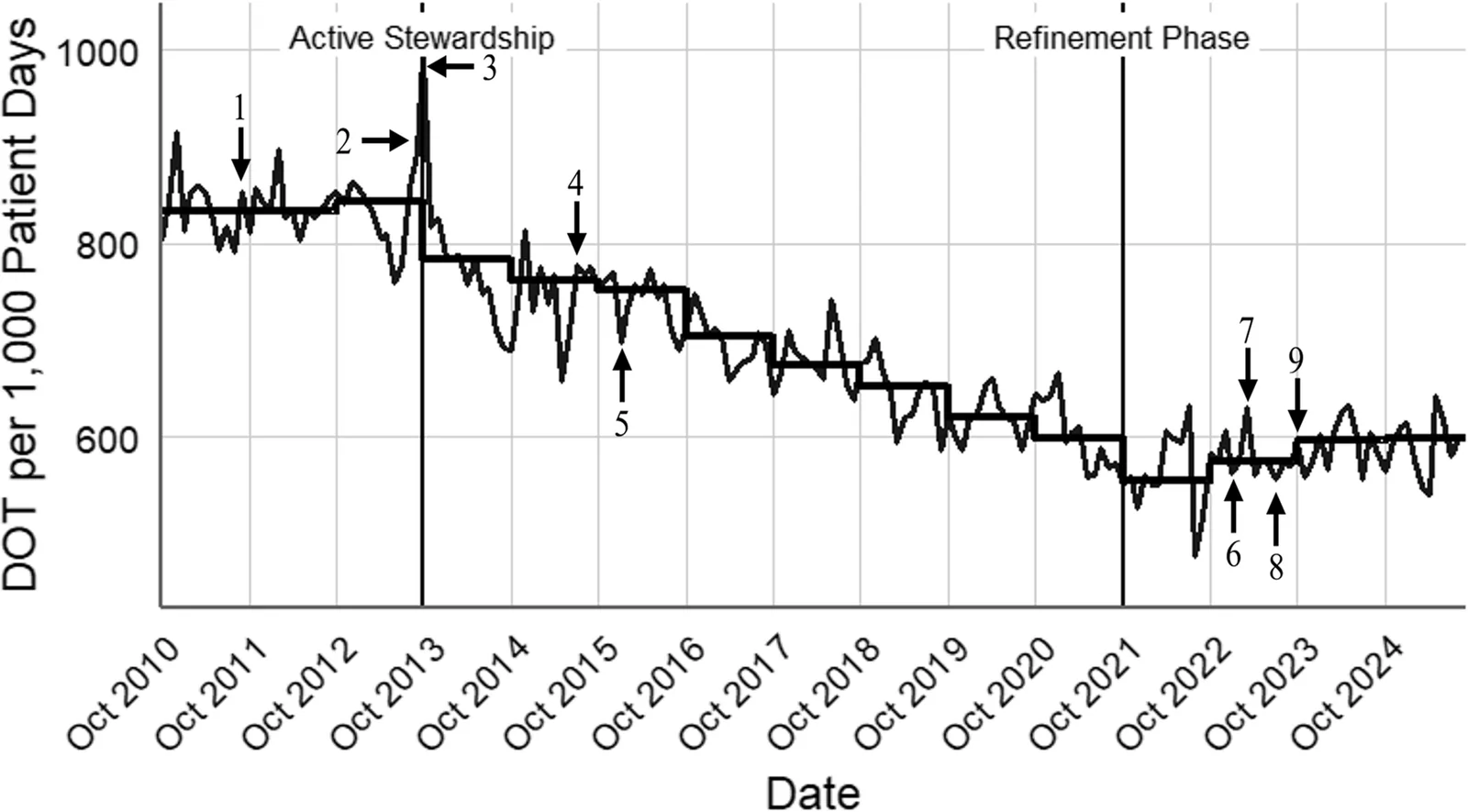
Antibacterial days of therapy (DOT) with yearly medians. *Note:* Arrows indicate milestones within the ASP program history: 1. ASP began creating institutional guidelines—September 2011; 2. ASP rounding 3 days/week - September 2013; 3. Indications required on 13 anti-infectives—October 2013; 4. ASP rounding 5 days/week—July 2014; 5. Indications required for all anti-infectives—January 2016; 6. Inpatient immunocompromised infectious diseases service began rounding regularly—January 2023; 7. Pharmacists began rounding for the ASP on weekends—March 2023; 8. Oral anti-infectives began requiring end dates—July 2023; 9. Firstline mobile application launched—October 2023.

The refinement phase was isolated and further analyzed to determine ongoing change in anti-infective use. Pediatric GP-MRSA agents changed from an estimated DOT/1,000 PD of 82.4–70.8 (−14.1%, *P* < .001) (Figure 3). For pediatric BSHO agents, DOT/1,000 PD remained stable at 123.8–119.7 (−3.4%, *P* = .529) over the 4-year period. Individual antimicrobials with significantly decreased estimated slope change per month include amoxicillin, cefepime, ciprofloxacin, and vancomycin (Table 2). Significant increases were observed with amoxicillin-clavulanate, azithromycin, ceftazidime, cephalexin, ertapenem, levofloxacin, linezolid, metronidazole, and sulfamethoxazole-trimethoprim. Enteral administrations were 30.3% prior to the refinement phase (HS1–HS7), 31.7% during HS8, and 34.9% in HS12. The case mix index (CMI) prerefinement (HS1–7) averaged 1.45 and post (HS8–HS12) was 1.62. Our anti-infective AWP-based cost/1,000 PD in 2021, the year our refinement phase began (HS8), was $50,516.17 compared to $38,520.45 in 2025 (HS12).

**Figure 3.**
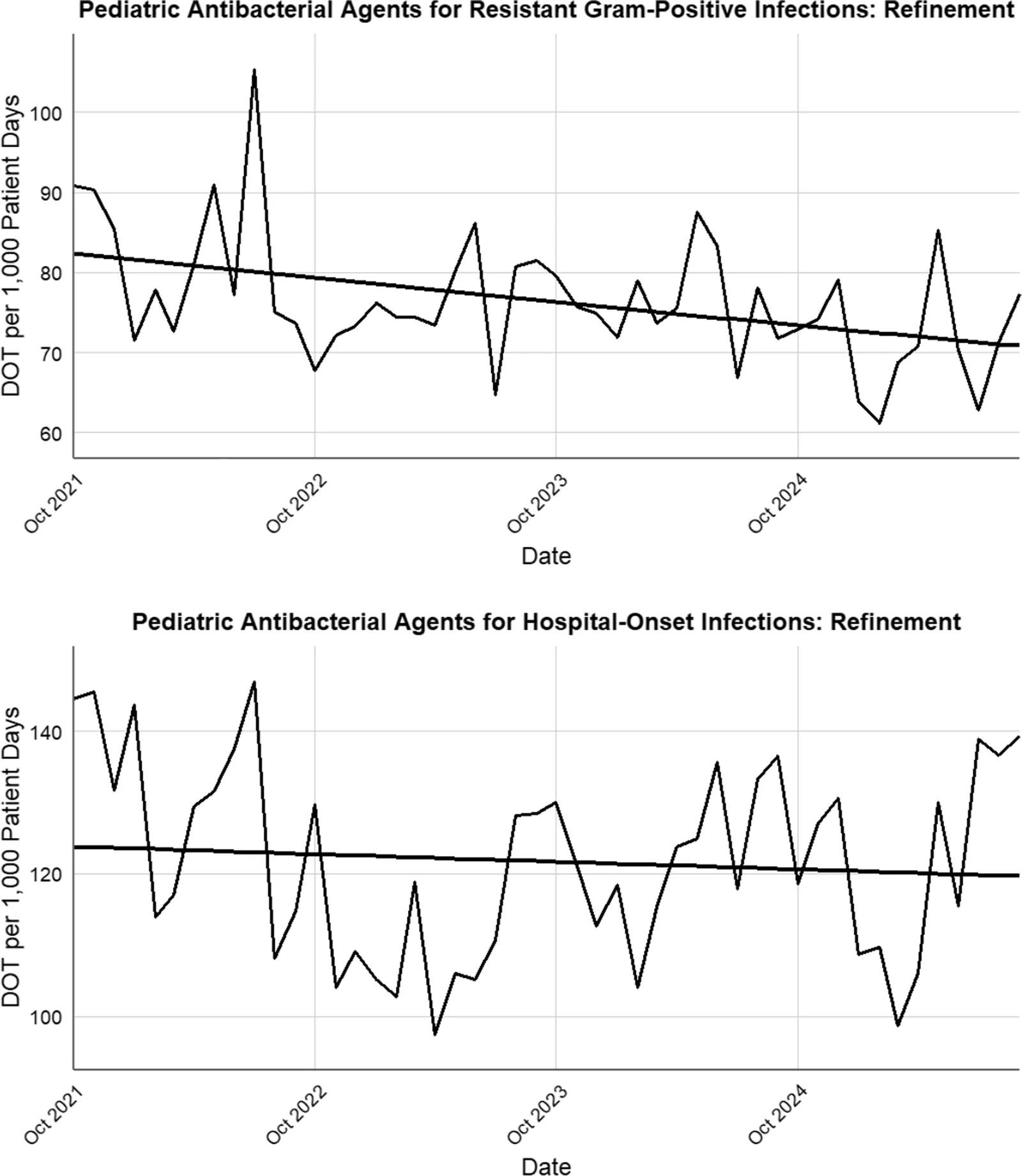
Figure 3 long description. Antibiotic days of therapy (DOT) in medication groupings for resistant gram-positive infections and hospital-onset infections during the refinement phase (October 2021–September 2025).

**Table 2.** Changes in days of therapy during the refinement phase by medication grouping and individual antibacterial (October 2021–September 2025), hospital-wide Table 2 long description.

Antibacterial agents	Percent change per month % (CI)	Regression coefficient *P*-value	Regression estimated absolute and percent change	Point estimates Oct 2021–Sept 2025 (DOT/1,000 PD)
Pediatric antibacterial agents for resistant gram-positive infections	−0.32% (−0.51, −0.13)	.001*	−11.6 (−14.1%)	82.4–70.8
Pediatric antibacterial agents for hospital-onset infections	−0.07% (−0.29, 0.15)	.529	−4.2 (−3.4%)	123.8–119.7
Amoxicillin	−1.05% (−1.56, −0.54)	<.001*	−9.5 (−39.1%)	24.2–14.8
Amoxicillin-clavulanate	+0.71% (0.03, 1.40)	.0352*	+11.6 (−39.4%)	29.3–40.9
Ampicillin	+0.20% (−0.17, 0.57)	.276	+3.9 (+10.0%)	39.3–43.2
Ampicillin-sulbactam	+0.15% (−0.26, 0.57)	.466	+1.6 (+7.5%)	21.4–23.0
Azithromycin	+1.91% (1.46, 2.37)	<.001*	+13.2 (+143.3%)	9.2–22.3
Cefazolin	+0.02% (−0.26, 0.29)	.896	+0.5 (+0.9%)	56.7–57.2
Cefepime	−0.31% (−0.60, −0.01)	.0437*	−8.4 (−13.4%)	62.4–54.1
Ceftaroline	−1.41% (−2.92, 0.13)	.0830	−1.2 (−48.6%)	2.5–1.3
Ceftazidime	+0.47% (0.09, 0.85)	.0173*	+4.4 (+24.6%)	18.0–22.5
Ceftriaxone	+0.13% (−0.17, 0.43)	.374	+3.3 (+6.4%)	51.1–54.4
Cephalexin	+0.52% (0.17, 0.87)	.0032*	+3.3 (+27.5%)	12.0–15.3
Ciprofloxacin	−1.41% (−2.10, −0.73)	<.001*	−3.3 (−48.8%)	6.7–3.4
Clindamycin	−3.49% (−4.12, −2.86)	<.001*	−11.2 (−81.2%)	13.8–2.6
Daptomycin	−0.11% (−1.64, 1.44)	.895	−0.1 (−5.2%)	1.8–1.7
Ertapenem	+4.72% (1.64, 7.85)	.00203*	+1.3 (+772.9%)	0.2–1.5
Gentamicin	+0.11% (−0.60, 0.83)	.758	+0.4 (+5.5%)	7.1–7.5
Levofloxacin	+0.80% (0.21, 1.40)	.00605*	+6.6 (+45.4%)	14.6–21.2
Linezolid	+3.01% (2.31, 3.71)	<.001*	+18.8 (+302.6%)	6.2–25.1
Meropenem	−0.40% (−1.12, 0.33)	.323	−1.6 (−17.1%)	9.5–7.9
Metronidazole	+0.96% (0.48, 1.44)	<.001*	+19.3 (+56.7%)	34.1–53.4
Piperacillin-tazobactam	−0.11% (−1.25, 1.04)	.867	−0.3 (−5.0%)	6.3–6.0
Sulfamethoxazole-trimethoprim	+0.41% (0.05, 0.77)	.0237*	+8.5 (+21.4%)	39.8–48.3
Vancomycin	−0.74% (−0.99, −0.49)	<.001*	−18.3 (−29.4%)	62.3–44.0

DOT, days of therapy; PD, patient days.

* Significant *P*-value (<.05).

## Discussion

Handshake stewardship is considered a leading practice in stewardship.^1,4^ However, as this method may require more dedicated resources, questions remain about whether it is worth the long-term investment; thus, outcomes after over a decade of work are important for programs weighing potential changes in stewardship design. At our institution, over a 12-year period of handshake stewardship, anti-infectives DOT/1,000 PD decreased by 33.0% (−330.3 DOT/1,000 PD, *P* < .001). These data build upon previous publications demonstrating efficacy during the postimplementation phase at our institution.^5,6^ A unique aspect of our handshake stewardship model is the lack of preauthorization and review of all anti-infectives. Many ASPs choose to review predefined broad-spectrum agents or restricted anti-infectives only; reviewing all anti-infectives allows our team an opportunity to teach stewardship concepts at every level. Through our daily conversations, we are easily able to find invested stakeholders to assess new guidelines and evaluate dogmatic practices, such as treatment of lymphadenitis, aspiration pneumonia, and surgical prophylaxis.^11–13^

As the goal of stewardship is judicious use, not no use, it is natural that decreases in use will slow and reach a maintenance/plateau phase, and gains will shift to appropriateness of agents: a refinement phase. As a new program in 2013, we had a lot of “low hanging fruit” to choose from; after twelve years, identifying areas for improvement and implementing (or de-implementing) change is more challenging. This more incremental use in appropriate choice of anti-infectives is observed during our refinement phase starting in October 2021 (HS8). For example, use of GP-MRSA agents continued to decrease overall, with decreasing vancomycin and other agents (eg, ceftaroline, clindamycin, daptomycin); though linezolid use increased, it did not replace the use of the other GP-MRSA agents. While use of pediatric BSHO agents remained steady, we made meaningful differences with certain agents. We targeted cefepime, ciprofloxacin, meropenem, and piperacillin-tazobactam through daily ASP rounds, which impacted the demonstrated declined prescribing. Increases were observed in ceftazidime, ertapenem, and levofloxacin during the refinement phase; while still comparatively low, we postulate these increases are in part due to loss of cefotaxime replaced by use of ceftazidime in neonates, use of levofloxacin for prophylaxis in the oncology population, and more recently, the use of ertapenem for synergy for *S. aureus* infections. Initiatives are underway to examine these trends, including assessment of the benefits versus risks of the use of levofloxacin prophylaxis, and pushing toward limited 24 hour empiric antimicrobials during sepsis evaluation for many populations.^14,15^ Maintenance of our intravenous to oral policy and encouragement on ASP rounds has likely led to significant increased use of enteral antibacterials, including striking changes in non-highly bioavailable agents (eg, amoxicillin instead of ampicillin). While changes in the proportions of highly bioavailable agents administered enterally weren’t demonstrated, this was influenced by overall decreased use of those agents, for example clindamycin.^16^ Our observed plateau is likely influenced by an increase in patient complexity when comparing the prerefinement phase to the refinement phase (HS 1–7 vs HS 8–12), indicated by a CMI increase of 0.17; while we considered adjusting for this factor, given the known limitations of CMI (such as billing practices), we did not.^17,18^

In judicious use, effective stewardship programs add value, for example better care for equal or less money. To assess value, it is thus important to think about cost and outcomes. Cost was evaluated our first year of handshake stewardship, where costs according to pharmacy purchasing data decreased from $10,545/1,000 PD during the planning period to $10,451/1,000 PD postASP; decreases in cost continued to be observed during our refinement phase, amounting to hundreds of thousands to millions in savings per year. ^19^ Clinical outcomes are arguably the most important result but much harder than DOT to measure, as clinical outcomes generally require carefully designed comparisons and chart review. That said, examples of clinical outcomes we evaluated include musculoskeletal infections, appendicitis, and acute otitis media, all more favorable in the handshake stewardship era.^20–23^ In addition, diagnostic errors were recognized by the stewardship team allowing real-time mitigation, and Infectious Diseases consults increased, which are associated with better outcomes.^24,25^

Areas of “high hanging fruit” for future focus will require in-depth review, stakeholder recruitment, and whole-system buy in. The surgical, hematology/oncology/BMT, and pediatric intensive care units are historically difficult areas to improve antimicrobial use; we have made progress but will continue targeted evaluation and work.^13,26,27^ For example, in oncology, the complex population and protocolized use makes abbreviated daily review less effective; thus, we are developing a dedicated immunocompromised stewardship service. Diagnostic stewardship has a domino effect on antimicrobial stewardship and thus this is an area where our team dedicates effort such as with blood cultures and tracheal aspirates.^28,29^ We continue to fine-tune and steward diagnostics as there are many opportunities to improve antimicrobial use in synergy with microbiology colleagues.^30,31^ For easy access to information, stewardship content related to pathways, diagnostics, pathogens, and antimicrobials was added to a freely available app (firstline.org/colorado).^32^ Additional future directions for us involve looking at cost savings of the program in-depth and further expansion of our ASP into the outpatient setting.

Strengths of this study include its wide lens, looking at nearly all units within Children’s Colorado, rather than just a subset, and the 12 years of data. The limited long-term ASP literature that has been published rarely exceeds a time line of over ten years and many studies tend to focus on specific units or agents.^33–38^ No long-term outcomes in handshake stewardship are reported, to our knowledge. Our study has a few limitations to consider. This study was completed at a single center which limits generalizability, and handshake stewardship is likely uniquely implemented. While we would like to report *Clostridioides difficile* and resistance data, the complexity of testing changes makes these data too flawed. Outcomes like length of stay, readmissions, and mortality are difficult to normalize to patient population over a 12-year span of time (with increasing CMI) and are impacted by many factors beyond antibiotic utilization that make them less useful in the context of this study. Similarly, anti-infective costs are difficult to attribute just to stewardship endeavors without accounting for healthcare inflation and changes in medication costs over time. Lastly, we have likely experienced increased variation in handshake stewardship practice with more clinicians and pharmacists in our ASP.

Though it may require more time, our program more than pays for itself, adding value in terms of better care for less money. Our long-term outcomes are encouraging in that antimicrobial stewardship trends and successes can be maintained without further additional resources. We have optimized our utilization of anti-infectives, and we continue to look for ways to refine our use. Enhancement of our stewardship program by looking at our outcomes, cost savings, and interventions, and focusing on areas with room for improvement including the intensive care and hematology/oncology/BMT units and outpatient services will further improve our reach. In conclusion, the handshake stewardship program at Children’s Colorado is both effective and sustainable; more publications in handshake stewardship in other settings would solidify this method as a leading best practice.^4^

## Figure 1. Long description

The image contains four line graphs showing the days of therapy (DOT) per 1,000 patient days for different types of anti-infective therapies from October 2010 to September 2025. Panel A: The graph titled ’Anti-infective Days of Therapy‘ shows the DOT per 1,000 patient days on the vertical axis and the date on the horizontal axis. The graph indicates a general downward trend in DOT over time, with a notable decrease after the implementation of active stewardship. Panel B: The graph titled ’Antibacterial Days of Therapy‘ also shows the DOT per 1,000 patient days on the vertical axis and the date on the horizontal axis. This graph similarly shows a downward trend in DOT, with a significant drop after active stewardship begins. Panel C: The graph titled ’Antifungal Days of Therapy‘ displays the DOT per 1,000 patient days on the vertical axis and the date on the horizontal axis. The trend in this graph shows a more variable pattern but also an overall decrease in DOT over time, particularly after active stewardship. Panel D: The graph titled ’Antiviral Days of Therapy‘ shows the DOT per 1,000 patient days on the vertical axis and the date on the horizontal axis. This graph exhibits fluctuations in DOT, with a general downward trend post-active stewardship.

Navigate back to Figure 1..

## Table 1. Long description

A table with 18 rows and 5 columns comparing changes in anti-infective use during a stewardship era. Columns include Stewardship slope change per month, Regression coefficient P-value, Regression estimated absolute and percent change, and Point estimates first to most recent year, DOT/1,000 PD. Row labels include various medication groupings and individual agents such as Anti-infectives, Antibacterials, Antifungals, Antivirals, Vancomycin, Linezolid, Clindamycin, Carbapenems, Fluoroquinolones, Ceftriaxone, Antipseudomonal cephalosporins, Piperacillin-tazobactam, and units like CICU/CPCU, NICU, PICU, Surgery/Rehabilitation, Hematology/Oncology/BMT, and General medicine. Each row provides specific data points for the respective medication or unit.

Navigate back to Table 1..

## Figure 3. Long description

Two line graphs depict antibiotic days of therapy (DOT) for pediatric antibacterial agents. Panel A: The line graph shows DOT per 1,000 patient days for resistant gram-positive infections from October 2021 to September 2025. The x-axis represents the date, and the y-axis represents DOT per 1,000 patient days. The graph shows fluctuations in DOT with a general downward trend over time. Panel B: The line graph shows DOT per 1,000 patient days for hospital-onset infections from October 2021 to September 2025. The x-axis represents the date, and the y-axis represents DOT per 1,000 patient days. The graph shows fluctuations in DOT with a relatively stable trend over time.

Navigate back to Figure 3..

## Table 2. Long description

A table comparing the percent change per month, regression coefficient P-value, regression estimated absolute and percent change, and point estimates from October 2021 to September 2025 for various antibacterial agents. The table has 21 rows and 4 columns. Column headers are: Antibacterial agents, Percent change per month % (CI), Regression coefficient *P*-value, Regression estimated absolute and percent change, Point estimates Oct 2021-Sept 2025 (DOT/1,000 PD). Row 1: Pediatric antibacterial agents for resistant gram-positive infections, −0.32% (−0.51, −0.13), .001*, −11.6 (−14.1%), 82.4−70.8. Row 2: Pediatric antibacterial agents for hospital-onset infections, −0.07% (−0.29, 0.15), .529, −4.2 (−3.4%), 123.8−119.7. Row 3: Amoxicillin, −1.05% (−1.56, −0.54), <.001*, −9.5 (−39.1%), 24.2−14.8. Row 4: Amoxicillin-clavulanate, +0.71% (0.03, 1.40), .0352*, +11.6 (−39.4%), 29.3–40.9. Row 5: Ampicillin, +0.20% (−0.17, 0.57), .276, +3.9 (+10.0%), 39.3–43.2. Row 6: Ampicillin-sulbactam, +0.15% (−0.26, 0.57), .466, +1.6 (+7.5%), 21.4–23.0. Row 7: Azithromycin, +1.91% (1.46, 2.37), <.001*, +13.2 (+143.3%), 9.2–22.3. Row 8: Cefazolin, +0.02% (−0.26, 0.29), .896, +0.5 (+0.9%), 56.7-57.2. Row 9: Cefepime, −0.31% (−0.60, −0.01), .0437*, −8.4 (−13.4%), 62.4−54.1. Row 10: Ceftaroline, −1.41% (−2.92, 0.13), .0830, −1.2 (−48.6%), 2.5−1.3. Row 11: Ceftazidime, +0.47% (0.09, 0.85), .0173*, +4.4 (+24.6%), 18.0–22.5. Row 12: Ceftriaxone, +0.13% (−0.17, 0.43), .374, +3.3 (+6.4%), 51.1–54.4. Row 13: Cephalexin, +0.52% (0.17, 0.87), .0032*, +3.3 (+27.5%), 12.0–15.3. Row 14: Ciprofloxacin, −1.41% (−2.10, −0.73), <.001*, −3.3 (−48.8%), 6.7−3.4. Row 15: Clindamycin, −3.49% (−4.12, −2.86), <.001*, −11.2 (−81.2%), 13.8−2.6. Row 16: Daptomycin, −0.11% (−1.64, 1.44), .895, −0.1 (−5.2%), 1.8−1.7. Row 17: Ertapenem, +4.72% (1.64, 7.85), .00203*, +1.3 (+772.9%), 0.2–1.5. Row 18: Gentamicin, +0.11% (−0.60, 0.83), .758, +0.4 (+5.5%), 7.1–7.5. Row 19: Levofloxacin, +0.80% (0.21, 1.40), .00605*, +6.6 (+45.4%), 14.6–21.2. Row 20: Linezolid, +3.01% (2.31, 3.71), <.001*, +18.8 (+302.6%), 6.2–25.1. Row 21: Meropenem, −0.40% (−1.12, 0.33), .323, −1.6 (−17.1%), 9.5−7.9.

Navigate back to Table 2..
